# IgA2 immune complexes selectively promote inflammation by human CD103^+^ dendritic cells

**DOI:** 10.3389/fimmu.2023.1116435

**Published:** 2023-03-16

**Authors:** Lynn Mes, Ulrike Steffen, Hung-Jen Chen, Jennifer Veth, Willianne Hoepel, Guillermo Romeo Griffith, Georg Schett, Jeroen den Dunnen

**Affiliations:** ^1^ Center for Experimental and Molecular Medicine, Amsterdam University Medical Centers (UMC), University of Amsterdam, Amsterdam Infection and Immunity Institute, Amsterdam, Netherlands; ^2^ Department of Medical Microbiology, Amsterdam University Medical Centers (UMC), University of Amsterdam, Amsterdam Infection and Immunity Institute, Amsterdam, Netherlands; ^3^ Department of Internal Medicine 3, Friedrich-Alexander-University Erlangen-Nürnberg and Universitätsklinikum Erlangen, Erlangen, Germany; ^4^ Department of Experimental Immunology, Amsterdam University Medical Centers (UMC), University of Amsterdam, Amsterdam Infection and Immunity Institute, Amsterdam, Netherlands; ^5^ Department of Rheumatology and Clinical Immunology, Amsterdam University Medical Centers (UMC), Amsterdam Rheumatology and Immunology Center, Amsterdam, Netherlands; ^6^ Department of Medical Biochemistry, Amsterdam University Medical Centers (UMC), University of Amsterdam, Amsterdam Infection and Immunity Institute, Amsterdam, Netherlands

**Keywords:** IgA subclasses, inflammation, intestine, CD103^+^ DCs, FcαRI

## Abstract

While immunoglobulin A (IgA) is well known for its neutralizing and anti-inflammatory function, it is becoming increasingly clear that IgA can also induce human inflammatory responses by various different immune cells. Yet, little is known about the relative role of induction of inflammation by the two IgA subclasses i.e. IgA1, most prominent subclass in circulation, and IgA2, most prominent subclass in the lower intestine. Here, we set out to study the inflammatory function of IgA subclasses on different human myeloid immune cell subsets, including monocytes, and *in vitro* differentiated macrophages and intestinal CD103^+^ dendritic cells (DCs). While individual stimulation with IgA immune complexes only induced limited inflammatory responses by human immune cells, both IgA subclasses strongly amplified pro-inflammatory cytokine production upon co-stimulation with Toll-like receptor (TLR) ligands such as Pam3CSK4, PGN, and LPS. Strikingly, while IgA1 induced slightly higher or similar levels of pro-inflammatory cytokines by monocytes and macrophages, respectively, IgA2 induced substantially more inflammation than IgA1 by CD103^+^ DCs. In addition to pro-inflammatory cytokine proteins, IgA2 also induced higher mRNA expression levels, indicating that amplification of pro-inflammatory cytokine production is at least partially regulated at the level of gene transcription. Interestingly, cytokine amplification by IgA1 was almost completely dependent on Fc alpha receptor I (FcαRI), whilst blocking this receptor only partially reduced cytokine induction by IgA2. In addition, IgA2-induced amplification of pro-inflammatory cytokines was less dependent on signaling through the kinases Syk, PI3K, and TBK1/IKKϵ. Combined, these findings indicate that IgA2 immune complexes, which are most abundantly expressed in the lower intestine, particularly promote inflammation by human CD103^+^ intestinal DCs. This may serve an important physiological function upon infection, by enabling inflammatory responses by this otherwise tolerogenic DC subset. Since various inflammatory disorders are characterized by disturbances in IgA subclass balance, this may also play a role in the induction or exacerbation of chronic intestinal inflammation.

## Introduction

Immunoglobulin A (IgA) is the most abundantly produced antibody of the human immune system (1–4). The majority of IgA is secreted at mucosal surfaces such as the intestine and the airways (5–7). In addition, IgA is the second most abundant antibody in serum (3). IgA has long been considered to be a non-inflammatory regulator that mostly counteracts infections by neutralization of pathogens. Yet, more recently IgA has been identified to also exert several pro-inflammatory effector functions (8, 9). Most of these effector functions are induced by activation of Fc alpha receptor I (FcαRI), which is expressed by various immune cells including neutrophils, macrophages, monocytes, and different subsets of dendritic cells (DCs) (10–13). FcαRI induces inflammatory responses when activated by IgA immune complexes that are formed upon binding of IgA to their antigens, which can be pathogens, infected cells, and even auto- or tumor-antigens. Individual FcαRI activation can directly induce immune activation by inducing neutrophil cytotoxicity and neutrophil extracellular trap (NET) formation (14, 15). However, for most cell types FcαRI needs to synergize with pattern recognition receptors (PRRs) such as Toll-like receptors (TLRs) to induce strong inflammatory responses (16, 17). Co-activation of FcαRI and PRRs particularly amplifies the production of pro-inflammatory cytokines such as tumor necrosis factor (TNF), interleukin (IL)-1β, and IL-23 through different transcriptional, translational, and post-translational mechanisms in a variety of cells including intestinal DCs, macrophages, monocytes, and Kupffer cells (17–20).

There are two IgA subtypes, IgA1 and IgA2, which have different structural characteristics and distinct localization and functionality (3, 21, 22). While IgA1 is most abundant in circulation, IgA1 and IgA2 are more evenly distributed in mucosal tissues (21). At particular mucosal sites IgA2 is even the most prominent subclass, especially in the lower intestine where its breakdown is less efficient than that of IgA1 due to the structural differences (3, 22–24). Recent studies have started to investigate the potential differences in induction of inflammation by IgA1 and IgA2. IgA2 complexes can induce NET formation by neutrophils to a greater extent than IgA1 complexes (21). In addition, stimulation of macrophages with IgA2 immune complexes results in higher levels of pro-inflammatory cytokine production. These findings could be relevant in the context of autoimmunity, since in diseases such as rheumatoid arthritis, disease-specific IgA autoantibodies are strongly shifted towards IgA2, which is associated with higher disease activity (21, 25). However, previous studies have only focused on individual stimulation of cells with IgA1 or IgA2 immune complexes. Since IgA mostly recognizes foreign structures such as microorganisms, IgA immune complexes frequently activate immune cells through simultaneous activation of FcαRI and PRRs. Yet, it is still unknown whether IgA subclasses induce different levels of inflammation upon co-stimulation with PRRs, or whether these responses exemplify cell type- or tissue-specific immunity.

In this study, we set out to determine whether IgA subclasses differ in their capacity to induce inflammatory responses in different human myeloid immune cells upon co-stimulation with PRR ligands. We identified that IgA1 induces more pro-inflammatory cytokine production by monocytes, whereas IgA2 induces more inflammation by CD103^+^ DCs. While inflammatory responses by CD103^+^ DCs induced by IgA1 were fully dependent on FcαRI and kinases Syk, PI3K, and TBK1/IKKϵ, IgA2 only showed partial dependency, suggesting the partial involvement of another receptor on these cells. In summary, this study identified that IgA subclasses induce cell type-specific inflammatory responses, which may serve a physiological function by providing tissue-specific immunity, but may also contribute to increased inflammation in autoimmune diseases that are characterized by increased levels of IgA2.

## Methods

### Cell culture

Buffy coats from anonymous healthy donors that were subjected to informed consent were provided by Sanquin Blood Supplies. Monocytes were isolated using Lymphoprep (Stemcell Technologies) and positive selection with CD14 magnetic microbeads (Miltenyi) and LS MACS Cell Separation Columns (Miltenyi). Monocytes were cultured in 24-well culture plates for 7 days in Iscove’s Modified Dulbecco’s Medium (IMDM) (Gibco) containing 86 µg/ml Gentamycin (Gibco), and supplemented with 5% fetal bovine serum (FBS) (Capricorn) and 20 ng/ml recombinant human GM-CSF (Invitrogen) or 10% FCS, 20 ng/ml GM-CSF, 1 µM retinoic acid (Sigma Aldrich) and 2 ng/ml IL-4 (Miltenyi) for macrophages and CD103^+^ DCs, respectively. After 3 days, supplemented culture medium was refreshed. On day 7, cells were detached using TrypLE select (Gibco). Monocytes were used for stimulation experiments directly after MACS isolation.

### Cell stimulation

Cells were stimulated *in vitro* with pooled human serum IgA (MP Biomedicals), or IgA1 or IgA2 isolated from pooled human serum from healthy donors as previously described (21, 26). Briefly, total IgA was isolated from the serum using peptide M agarose (InvivoGen). IgA1 and IgA2 were separated by Jacalin-based chromatography (Thermo Scientific). Purity of the samples was checked by western blot and Coomassie assays. IgA preparations were devoided from LPS by Triton-X114 treatment (Sigma) as described by Steffen and colleagues (26). LPS removal was verified with a LAL (Limulus Amebocyte Lysate) chromogenic endotoxic quantitation test (Thermo Scientific).

For experiments assessing the influence of differential glycosylation on the inflammatory capacity of IgA subclasses, deglycosylation of IgA1 was performed using PNGaseF treatment as described by Steffen et al. (21). In short, 1000 U PNGaseF (NEB) was added to 1 mg IgA1 and incubated at 37°C for 18h. The deglycosylation was confirmed performing lectin blot assays with lens culinaris agglutinin (Vector Laboratories).

High affinity plates (Nunc MaxiSorp; ThermoFisher Scientific) were coated with total IgA (MP Biomedicals), IgA1 or IgA2 in a concentration of 4 µg/ml unless stated otherwise. After overnight incubation, plates were blocked for 1h at 37°C with PBS containing 10% FCS. For stimulation, cells were seeded in IgA-coated plates (40.000 cells/well) in the presence of 10 µg/ml Pam3CSK4 (InvivoGen), 100 ng/ml LPS (E. coli o111:B4; Sigma Aldrich), or 10 µg/ml PGN (S. aureus; Sigma Aldrich). To block FcαRI, cells were pre-incubated with 20 µg/ml anti-CD89 (MIP8a; MyBiosource) for 0.5h at 4°C. Before stimulation, cells were diluted in medium to an end concentration of 5 µg/ml antibody. For inhibitor treatment, cells were pre-incubated for 0.5h at 37°C with 0.5 µM R406 (MedChemExpress), 25 µM alpelisib (Selleckchem), 1 µM BX795 (InvivoGen), or DMSO (Merck).

### Enzyme linked immunosorbent assay

Cytokine production was analyzed after 24 hours stimulation. Cytokine levels in the supernatant were measured by Enzyme linked immunosorbent assay (ELISA) using IL-23 (U-CyTech Biosciences) and TNF (eBiosciences) antibody pairs.

### Meso Scale Discovery multiplex assay

Multiplexing human cytokine assay kit U-PLEX (IL-1β, IL-6, IL-8, IL-10, TNF, and CXCL10) were purchased from Meso Scale Discovery (MSD). The standards for Proinflammatory Panel 1 and Chemokine Panel were reconstituted in provided assay diluents. U-PLEX plates were coated with the linkers and biotinylated capture antibodies per the manufacturer’s protocol. The supernatant was collected after 24 hours stimulation. The electrochemiluminescence signal was detected by a MESO QuickPlex SQ 120 plate reader (MSD) and analyzed with Discovery Workbench Software (v4.0, MSD). The concentrations were calculated based on the four-parameter logistic fitting model generated with the standards (standard concentrations were provided in the MSD certificate of analysis).

### Quantitative real-time PCR

After stimulation, cells were lysed at different time points (0, 1.5, 3, and 6h). RNA extraction was performed using RNeasy Mini kit (Qiagen) according to manufacturer’s protocol. For cDNA synthesis a High-Capacity cDNA Reverse Transcription Kit (Applied Biosystems, ThermoFisher Scientific) was used following manufacturer’s instructions. qPCR was performed with a SensiFAST SYBR No-ROX Kit (Bioline, Meridian Bioscience) and primers as stated in **Table 1** (Sigma Aldrich), and measured on the LightCycler 480 (Roche).

**Table 1:** Primer pairs for quantitative RT-PCR.

Target	Forward sequence	Reverse sequence
*CXCL8*	ATACTCCAAACCTTTCCACC	TCCAGACAGAGCTCTCTTCC
*FCAR*	ACGACGCAGAACTTGATCCG	ATGGCTGTGCCAATTTTCAAC
*HPRT1*	GACCAGTCAACAGGGGACAT	AACACTTCGTGGGGTCCTTTTC
*IL1B*	AAGATGCTGGTTCCCTGC	GTTCAGTGATCGTACAGGTGC
*IL6*	CCTGAACCTTCCAAAGATGGC	TTCACCAGGCAAGTCTCCTCA
*RACK1*	GAGTGTGGCCTTCTCCTCTG	GCTTGCAGTTAGCCAGGTTC
*TNF*	GGCGTGGAGCTGAGAGAT	TGGTAGGAGACGGCGATG
*UBB*	GGTCCTGCGTCTGAGAGGT	GCCTTCACATTTTCGATGGTGT

### Flow cytometry

After 7 days, differentiated DCs were harvested and stained with anti-CD103 (Ber-Act8; Biolegend) and anti-CD89 antibodies (A59; Biolegend). Samples were analyzed using flow cytometry (CytoFLEX, Beckman Coulter). Flowjo software v10.8 (BD Life Sciences) was used to analyze the data.

### Statistical analysis

Statistical analysis was performed with GraphPad Prism v9.1.0 for Windows (GraphPad Software). Two-way ANOVA tests (multiple comparisons with Sidak correction) were applied to cytokine production data and students t-tests were used to determine statistical significance of FcαRI blocking and inhibition of Syk, PI3K and TBK1/IKKϵ. Gene expression levels were analyzed by multiple t-testing. P-values <0.05 were considered significant and indicated as * or ** for p-values <0.05 and <0.01, respectively.

## Results

### IgA2 induces more pro-inflammatory cytokine production by CD103^+^ DCs than IgA1

**Figure 1:**
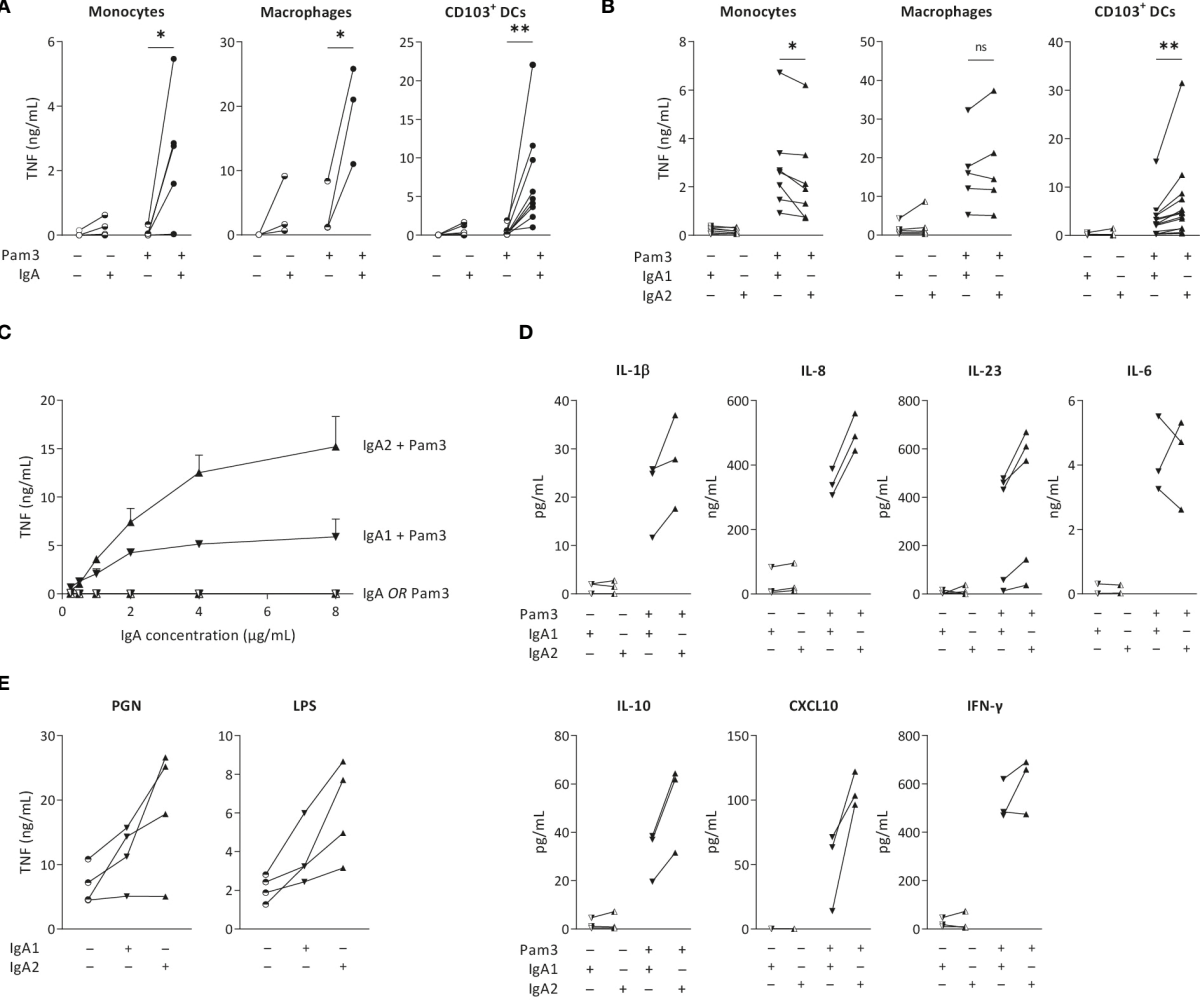
IgA2 immune complexes selectively increase cytokine production by intestinal CD103^+^ DCs. Monocytes, macrophages and CD103^+^ dendritic cells (DCs; 40,000 cells/well) were stimulated with TLR2/1 agonist Pam3CSK4 (Pam3) and serum isolated total IgA **(A)**, IgA1, or IgA2 **(B)**, and analyzed for pro-inflammatory cytokine production after 24 hours. TNF production by CD103^+^ DCs after (co)-stimulation with different concentrations of IgA subclasses was analyzed **(C)**. Production of different cytokines by CD103^+^ DCs upon (co-) stimulation was measured by Meso Scale Discovery multiplex assay **(D)**. TNF production upon alternative PRR stimulation with PGN or LPS was tested by ELISA **(E)**. Data of multiple donors **(A, B, D, E)** or a representative example measured in triplicate **(C)**. *p < 0.05; **p<0.01; ns – not significant.

To validate previous findings that IgA immune complexes can induce inflammatory responses by different human cell types, we stimulated monocytes, monocyte-derived GM-CSF-differentiated macrophages, and monocyte-derived CD103^+^ DCs, of which the latter closely resembles the CD103^+^ SIRPα^+^ subset of primary human intestinal DCs (19, 27, 28). IgA immune complexes were formed by immobilization of serum-purified IgA on high-affinity plates, which has previously been validated as a standardized method to reproducibly generate immune complexes that induce cytokine responses similar to immune complexes generated by opsonized bacteria (19, 29), cells expressing viral proteins (30), antibody-coated beads (31), and heat-aggregated antibodies (32). While individual stimulation with IgA immune complexes only induced low levels of the pro-inflammatory cytokine TNF by monocytes, macrophages and CD103^+^ DCs, IgA immune complexes strongly amplified pro-inflammatory cytokine production upon co-stimulation with TLR2/1 ligand Pam3CSK4 (**Figure 1A**), thereby confirming previous findings.

To determine the overall effect of IgA subclasses on the cellular inflammatory state, we purified IgA from pooled serum of healthy individuals and separated IgA1 from IgA2. Strikingly, while co-stimulation with IgA1 induced higher or similar levels of pro-inflammatory cytokines by monocytes and macrophages, respectively, IgA2 induced substantially more TNF than IgA1 by CD103^+^ DCs (**Figure 1B**). Since we observed the most pronounced difference between IgA1 and IgA2 in CD103^+^ DCs, we continued our research using this cell-type. To determine whether the difference between IgA subclasses is concentration dependent, we stimulated CD103^+^ DCs with increased concentrations of immobilized IgA. As shown in **Figure 1C**, IgA2 induced more TNF production in every tested concentration. Interestingly, while the curve for TNF production upon co-stimulation with IgA1 already flattens at 2 µg/ml of IgA, IgA2 still showed increased TNF production at 4 and 8 µg/ml of IgA, thereby suggesting qualitative differences between the two IgA subclasses (**Figure 1C**).

In addition to the classical pro-inflammatory cytokine TNF, we also assessed potential differences between IgA subclasses in production of other pro- and anti-inflammatory cytokines. In CD103^+^ DCs, IgA2 co-stimulation more strongly amplified the production of cytokines IL-1β, IL-8, IL-10, TNF, and CXCL10, than IgA1 (**Figure 1D**). In contrast, no clear differences were found for IL-6 and IFN-γ (**Figure 1D**). In addition to TLR2/1 ligand Pam3CSK4, IgA2 immune complexes also showed increased TNF production upon co-stimulation with LPS (TLR4 activation) and PGN (TLR2 and NOD-like receptor activation) (**Figure 1E**), indicating that IgA subclass-specific responses are induced upon co-stimulation with different PRRs.

### IgA2 induces higher levels of cytokine gene transcription than IgA1

**Figure 2:**
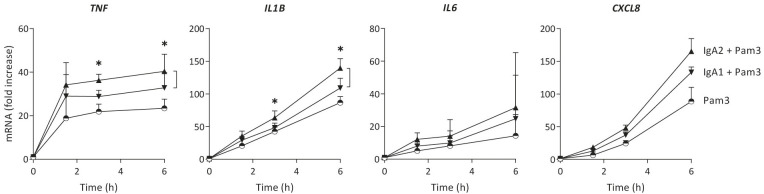
Increased cytokine production by IgA2 is at least partially mediated by enhanced gene transcription. mRNA expression of cytokines by CD103^+^ DCs (40,000 cells/well) upon stimulation by Pam3CSK4 (Pam3), or co-stimulation with IgA1 or IgA2 was measured at different time points. Expression levels of indicated genes were normalized to the mean expression of *RACK1*, *HPRT1*, and *UBB*. Data shown of four donors (mean + SD). *p < 0.05.

Cytokine production can be regulated at different levels. To determine whether IgA subclass-specific cytokine responses in CD103^+^ DCs are regulated at the level of gene transcription, we analyzed mRNA expression of the genes of interest over time. The relative expression levels of pro-inflammatory cytokines were in line with the protein data, as co-stimulation with IgA2 showed higher *TNF*, *IL1B* and *CXCL8* mRNA levels as compared to co-stimulation with IgA1 (**Figure 2**). In line with the protein data, *IL6* mRNA expression was increased upon IgA co-stimulation, but not different between the subclasses. These data indicate that differences in amplification of pro-inflammatory cytokines by IgA subclasses is regulated at least partially at the level of gene transcription.

### IgA2-induced cytokine production is only partially dependent on FcαRI

**Figure 3:**
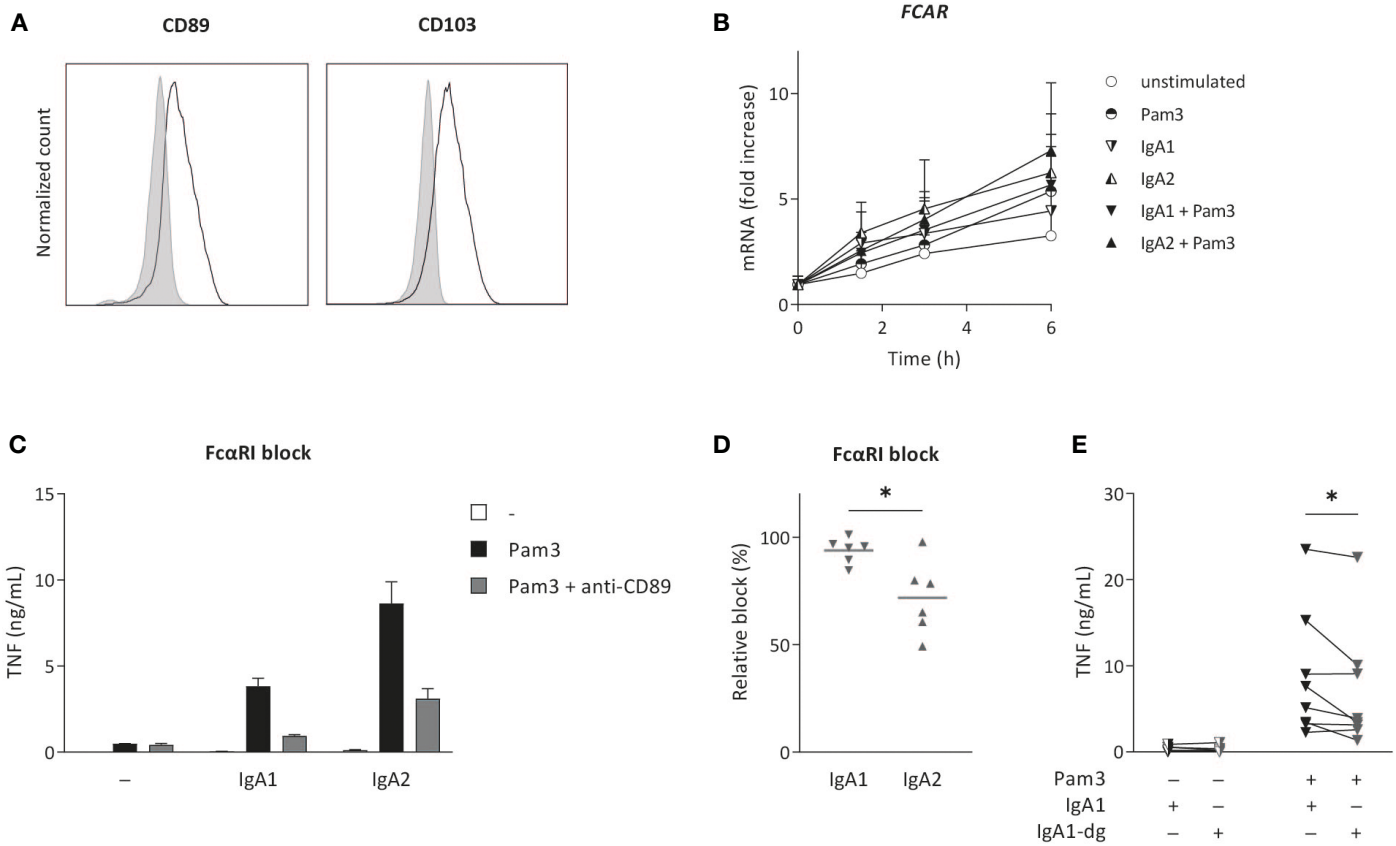
IgA2-induced cytokine production is only partially dependent on FcαRI. Surface expression of FCαRI (CD89) and CD103 was analyzed on *in vitro* differentiated CD103^+^ DCs **(A)**. Expression was compared to unstained cells (grey). Data shown of a representative example of four donors. *FCAR* mRNA expression after (co-) stimulation with Pam3CSK4 (Pam3) and IgA1 or IgA2 was measured at indicated time points and normalized to the mean expression of *RACK1*, *HPRT1*, and *UBB* (data of four donors shown as mean + SD) **(B)**. Pro-inflammatory cytokine production upon (co-) stimulation (40,000 cells/well) after pre-treatment with anti-CD89 antibodies (FcαRI block) was analyzed (representative example, measured in triplicate) **(C)**. The effect of anti-CD89 pre-treatment on TNF production was compared to co-stimulation of non-treated CD103^+^ DCs **(D)**. Data of six independent experiments. Effects of N-glycosylation on cytokine production was tested for multiple donors using de-glycosylated IgA1 (IgA1-dg) in stimulation experiments with Pam3 **(E)**. *p < 0.05.

The main receptor for IgA on human myeloid cells is FcαRI (CD89) (33), which was expressed on the cell surface of CD103^+^ DCs (**Figure 3A**). During (co-) stimulation, FcαRI gene expression (*FCAR*) increased over time to a minor extent, but no major differences were observed between stimulation with either IgA1 or IgA2 (**Figure 3B**). To assess the dependency of cytokine induction by CD103^+^ DCs of the IgA subclasses, we blocked FcαRI using a specific antagonistic antibody and analyzed cytokine production. Blocking of the FcαRI dampened TNF production induced by co-stimulation with IgA1 or IgA2 immune complexes without affecting Pam3CSK4-induced cytokine production (**Figure 3C**). Interestingly, while cytokine production induced by IgA1 was almost fully dependent on the FcαRI, blocking the receptor only lead to a partial reduction of IgA2-induced TNF production (**Figures 3C–D**).

The difference in inflammatory capacity of IgA subclasses upon individual stimulation (i.e. in absence of co-stimulation with PRR ligands) has previously been attributed to differences in their glycosylation profile (21). More specifically, higher levels of sialic acid in IgA1 glycans were shown to be responsible for its reduced induction of inflammation, and removal of N-glycans of IgA1 increased neutrophil and macrophage activation to the level of IgA2 (21). To determine whether this difference in IgA1 glycosylation is also responsible for reduced inflammation upon co-stimulation of CD103^+^ DCs, we compared cytokines induced by native IgA1 and IgA1 of which N-glycans (including sialic acid) were removed. Interestingly, removal of N-glycans from IgA1 did not increase IgA1-induced cytokine production by CD103^+^ DCs, but even slightly decreased pro-inflammatory cytokine production (**Figure 3E**). This indicates that, in contrast to individual stimulation of neutrophils and macrophages, the reduced inflammatory capacity of IgA1 upon co-stimulation of CD103^+^ DCs is not dependent on the increased expression of N-linked sialic acid.

### IgA2-induced cytokine production is less dependent on Syk, PI3K, and TBK1/IKKϵ than IgA1

**Figure 4:**
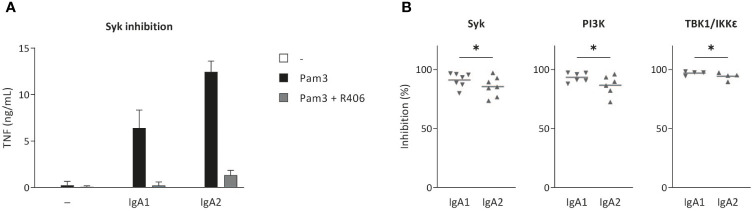
IgA-induced cytokine production can be abolished by small molecule inhibitors targeting downstream signaling molecules for FcαRI. Cytokine production by CD103^+^ DCs (40,000 cells/well) upon co-stimulation with Pam3CSK4 and IgA1 or IgA2 after treatment with small molecule inhibitors R406 **(A, B)**, alpelisib, or BX795 **(B)** specific for Syk, PI3K and TBK1/IKKϵ, respectively, was measured by ELISA. TNF production of cells pre-treated with inhibitors was normalized to that of DMSO controls. Data of a representative example measured in triplicate (mean + SD) **(A)** or multiple donors **(B)**. *p < 0.05.

Next, we aimed to identify whether inflammation induced by co-stimulation of CD103^+^ DCs with IgA1 or IgA2 immune complexes is dependent on the same signal transduction pathways. FcαRI-induced inflammation by human CD103^+^ DCs is known to critically depend on signaling through kinases Syk, PI3K, and TBK1/IKKϵ (19). Inhibition of Syk by the specific inhibitor R406 inhibited both IgA1- and IgA2-induced TNF production by CD103^+^ DCs (**Figures 4A, B**). Yet, IgA2-induced TNF production showed reduced dependency on Syk compared to IgA1 (**Figure 4B**). Similarly, IgA2-induced inflammatory responses were less dependent on PI3K (inhibited by alpelisib) and TBK1/IKKϵ (inhibited by BX795) (**Figure 4B**). These data indicate that IgA2-induced inflammatory responses by co-stimulation of CD103^+^ DCs are partially independent on FcαRI signaling kinases Syk, PI3K, and TBK1/IKKϵ.

## Discussion

Immunoglobulins are essential to protect our body from invading pathogens, which they mediate through a variety of effector functions, such as neutralization, phagocytosis, and the induction of inflammation. While IgG subclasses (IgG1-4) have long been known to differently activate antibody effector functions, and to thereby tailor immune responses to the situation at hand (16, 34, 35), less is known about the differences in immune activation by IgA subclasses. Here, we provide evidence that human IgA also induces subclass-specific immunity, which mediates cell type-specific inflammatory responses. While IgA1, the main subclass in circulation, induces slightly higher pro-inflammatory cytokine production by monocytes, IgA2, which is more prevalent in the lower intestine, selectively promotes inflammation by human CD103^+^ intestinal DCs. Interestingly, the inflammatory response of CD103^+^ DCs induced by IgA2 immune complexes is only partially dependent on FcαRI and its signaling molecules Syk, PI3K, and TBK1/IKKϵ, suggesting the involvement of another IgA2-sensing receptor. Together, these findings suggest that IgA can induce subclass-specific immune responses that could contribute to orchestration of tissue-specific immunity.

Humans are one of the few species that express two IgA subclasses (2, 15). For example, mice only express one IgA isotype and do not express FcαRI (12, 36), which combined has likely hampered the study of potential IgA subclass-specific immune responses. Recent studies that have started to investigate the differences in effector functions of the IgA subclasses mostly focused on the effect of individual stimulation of immune cells with IgA immune complexes. While individual stimulation is sufficient for activating particular IgA effector functions such as NETosis by neutrophils, it is insufficient for inducing robust pro-inflammatory cytokine production. IgA immune complexes induce pro-inflammatory cytokine production by amplifying the response of PRRs, which typically occurs when immune cells encounter IgA-opsonized microorganisms (9). Notably, IgA2 seems more pro-inflammatory than IgA1 upon both individual stimulation (of neutrophils and M-CSF-differentiated macrophages), as previously shown by Steffen et al. (21), and co-stimulation with PRR ligands (of CD103^+^ DCs), as we describe in this study. However, our data indicate that it would be an oversimplification to generally categorize IgA2 as the more inflammatory IgA subclass. For example, co-stimulation with IgA1 immune complexes induced more pro-inflammatory cytokine production than IgA2 by human monocytes, while we observed no differences upon co-stimulation of (GM-CSF-differentiated) macrophages. Only for human CD103^+^ DCs we observed substantially higher inflammatory responses induced by IgA2. This cell type-specific inflammatory capacity of the IgA subclasses may therefore serve an important physiological role by contributing to the induction of context-specific immunity, i.e. tailoring of immune responses to the situation at hand. In addition to that, the known different antigen-specificities of IgA1 and IgA2 (37–39) will further contribute to pathogen-specific immunity.

Human CD103^+^ DCs, the main cell type that showed increased responsivity to IgA2, reside in the lamina propria of the intestine. These cells are relatively likely to encounter IgA immune complexes because of the high presence of microorganisms in the intestine. The IgA in the lamina propria is produced by local plasma cells, and is tailored to the individual’s microbiota, particularly to bacterial species that are colitogenic (4, 40, 41). As soon as bacteria penetrate the epithelial cell layer, IgA-opsonized bacteria form immune complexes that are recognized by FcαRI-expressing immune cells such as CD103^+^ DCs. While bacteria in the lumen are generally opsonized by secretory IgA that a has reduced affinity for FcαRI (12, 21, 42), invading bacteria are likely to be (also) opsonized by conventional dimeric IgA that is present at high levels in the lamina propria, leading to FcαRI activation. A limitation of our study is that for our *in vitro* stimulation assays we have used serum IgA, which is monomeric, while locally produced IgA in the lamina propria is mainly dimeric (43). Yet, since the affinity of monomeric and dimeric IgA immune complexes for FcαRI does not differ substantially (44), and we have previously shown that monomeric and dimeric IgA are identical in their capacity to induce cytokines (18), the observed differences in IgA subclass-specific responsivity are expected to be similar for dimeric IgA.

Remarkably, our current finding of increased inflammatory responses of CD103^+^ DCs upon stimulation with IgA2 is in disagreement with our previous work, in which we did not find differences between IgA1 and IgA2 (19). This is likely to be caused by the source of the IgA subclasses, since in our previous work we used recombinant IgA, while for the current study we used IgA that was purified from human serum. Recombinantly produced IgA generally displays less variety and different post-translational modifications (such as glycosylation) as compared to human serum IgA. Since serum IgA better reflects the human physiological situation than recombinantly produced IgA, human serum IgA2 is most likely to induce increased inflammatory responses *in vivo*.

IgA2 immune complexes induced increased pro-inflammatory cytokine gene transcription by CD103^+^ DCs compared to IgA1. This is adding another layer of amplification of inflammation by CD103^+^ DCs, since previously we had already shown that IgA immune complexes amplify cytokine production through increased gene translation and caspase-1 activation (19). Of note, CD103^+^ DCs are generally tolerogenic cells, which show little response to individual stimulation with PRR ligands (19). IgA1 immune complexes are known to convert this tolerogenic response, whilst IgA2 immune complexes are able to further amplify this inflammatory response through another mechanism that acts at the level of gene transcription.

Interestingly, while IgA1-induced cytokine production was completely dependent on FcαRI, IgA2-induced inflammation only showed partial (~70%) dependency on this receptor. This suggests that co-stimulation with IgA2 immune complexes involves the activation of another receptor in addition to FcαRI. Although IgA2-induced cytokine production was also slightly (~5%) less dependent on FcαRI signaling molecules Syk, PI3K, and TBK1/IKKϵ, these differences were smaller than the difference in FcαRI dependency (compare **Figures 3D**, **4B**). Although dependency on Syk signaling for cytokine induction by IgA1 and IgA2 is only slightly different, this difference is statistically significant and may be of importance to the molecular mechanism underlying the observed effect induced by the different IgA subclasses. The identity of the additional (co-) receptor (or receptors) is still unknown, but considering the previously identified differences in glycosylation between IgA subclasses (21), it is tempting to speculate about a potential role of carbohydrate-sensing receptors such as C-type lectins. Myeloid immune cells express a variety of C-type lectins (45, 46), some of which also signal through Syk (47–49). For future research, studying the involvement of C-type lectins expressed by CD103^+^ DCs may provide a first step to identify the receptor responsible for IgA2-induced inflammation. In addition, it would be interesting to explore the potential involvement of extracellular signal-regulated kinase (ERK), since in neutrophils ERK is activated by IgA2, but not IgA1 (21).

Although IgA subclass-specific immune responses most likely serve a physiological function by enabling tissue-specific immunity, it may also lead to adverse events by undesirably promoting inflammation. Several autoimmune diseases are characterized by IgA autoantibodies (8). For example, specific autoantibodies for rheumatoid arthritis (RA) are autoantibodies against citrullinated protein antigens (ACPA), which are also of the IgA isotype (50). Notably, in serum of RA patients the IgA1:IgA2 ratio of ACPA is strongly shifted towards IgA2, which correlates with disease activity (21). For future studies, it would be interesting to assess whether synovial macrophages of RA patients similarly show increased responsivity towards IgA2, which may explain the correlation of IgA2 and higher disease scores. Interestingly, the frequently observed intestinal symptoms in RA patients (51–53) are in line with the increased responsivity of the intestinal immune system to IgA2. Also in celiac disease, characterized by high levels of IgA against both gluten and the autoantigen transglutaminase 2 (54), IgA2 levels are associated with disease severity (55).

Since also bronchial mucosa is at baseline slightly enriched for IgA2 (5), investigation of immune responsivity to IgA2 may be of interest for conditions that manifest in the airways. Interestingly, allergen-specific immunotherapy has been found to induce specifically IgA2 antibody production in individuals with airway allergies (56), suggesting immune suppressing effects in this context. In contrast, IgA2 may also play a role in exacerbation of inflammation in infectious diseases such as COVID-19, where IgA2 levels correlate with fatal outcome in severely ill patients (57). In addition, IgA deficiency has been previously linked to increased susceptibility to chronic inflammatory conditions (58), indicating the complex role of IgA in mucosal immunity.

Taken together, these results indicate that IgA subclasses induce cell type-specific immune responses upon co-stimulation with PRR ligands, with IgA2 inducing particularly pronounced inflammatory responses by CD103^+^ DCs. While its physiological function is most likely to provide tissue-specific immunity, it may also contribute to excessive inflammation in diseases associated with increased levels of IgA2, such as RA, celiac disease, and COVID-19.

## Data availability statement

The raw data supporting the conclusions of this article will be made available by the authors, without undue reservation.

## Author contributions

Conceptualization: JD. Methodology: LM, JD, WH, H-JC. Formal analysis: LM. Investigation: LM, JV, US, H-JC, GRG. Writing original draft: JD, LM. Review & editing: JD, LM, US, H-JC, WH, GS. All authors contributed to the article and approved the submitted version.
